# New Nordic Nutrition Recommendations are here

**DOI:** 10.3402/fnr.v57i0.22903

**Published:** 2013-10-03

**Authors:** Mikael Fogelholm

**Affiliations:** University of Helsinki, Department of Food and Environmental Sciences

Nordic countries have a long tradition of developing joint nutrition recommendations. The latest recommendations, issued on 3 October 2013 (called the Nordic Nutrition Recommendations, NNRs, 2012) are already in their fifth edition. The first NNRs were released in 1980 and thereafter a new edition has been issued every eight years. While the 1996 edition was published in Swedish, the two more recent recommendations have can be read in English. This reflects a recognition that the scientific basis for nutrition recommendations is not limited to specific countries, but should be of interest to a wider group of experts and decision makers.

During recent years, interest in as well as critique of nutrition recommendations has been stronger than ever. In light of this, the NNR project group decided early on to use systematic reviews (SR) as a base for several of the chapters with nutrient or food-based recommendations. *Food & Nutrition Research* was selected as an outlet for the SRs in the project. An important argument behind this decision was to ensure high quality reviews and open access to the published works, such that the recommendations would be freely available to all interested parties.

During the year 2012, Food & Nutrition Research published SRs on dairy consumption and fetal growth (1), diet and long-term weight change (2), iodine (3), and sugar (4). These four have been followed in 2013 by SRs on iron (5), weight loss before conception (6), breastfeeding (7), protein intake during childhood and adolescence (8), dietary fiber and glycaemic index (9), protein intake in adults (10), calcium (11), and foods and dietary patterns (12). Still awaiting publication are reviews on, for example, vitamin D and fatty acids, which are two extremely ‘hot’ topics. In addition to informing the NNR, these articles constitute a unique collection of quality reviews that should be of interest to anyone seeking up-to-date information on the relationships between nutrients and food and health.

I have heard a number of comments on what are perceived to be small or non-existent changes in the most recent NNR, compared to the previous edition. However, a certain degree of stability in the recommendations is in fact expected for two reasons. First, since the recommendations are based on the ‘totality of evidence’; that is, cumulative knowledge, a few new publications will seldom contain enough new knowledge to overthrow earlier views. Second, arriving at NNR is a consensus process. Radical changes in recommendations would only be possible if the process was carried out by a few radical individuals. In fact, about 100 scientists from all five Nordic countries were involved in developing the present recommendations, making radical shifts unlikely.

Nevertheless, some changes are noteworthy in this edition. These reflect shifts in knowledge with roots in 1996 to 2004 and later 2008. In 1996 (13), the recommendation for total fat intake was ‘not more than 30% in total energy intake (E%)’. In 2004 (14), the recommendation was given as a range (25–30 E%), and 30 E% (precisely, instead of less than) was given as the population planning. In the most recent edition, the recommended range is 25–40 E% and for planning 32–33 E%. This is an interesting trend towards gradually loosening restrictions in total fat intake. Looking at the changes in different types of fats, we see that the increase in total fat intake is associated mainly with an increased upper limit for mono-unsaturated fatty acids. I would no longer call the NNRs a ‘recommendation for low-fat intake’ – although this is still a misconception many (lay) individuals seem to have.

The second shift relates to vitamin D: the recommendation for adults has increased from 5 mg in 1996 to 7.5 mg in 2004 and now to 10 mg in the new edition. If doubling the recommended intake in 16 years is not a significant change, what is? The revised view on vitamin D recommendations demonstrates that if the evidence is available, researchers are willing to accept new conclusions.

Developing the new NNRs has been a tremendous task. With the growing body of research, one can ask whether the task of reviewing evidence has grown beyond the efforts of five small countries to catalogue. Would it make sense to consider working together across a larger group of nations, at least with respect to nutrient recommendations (if not food-based dietary guidelines)? Should all European countries work together? Regardless of how and where future recommendations will be developed, the current NNR offer highly valuable information, based on thorough reviews that are freely available here in Food & Nutrition Research.

*Mikael Fogelholm*, Professor in Nutrition University of Helsinki, Department of Food and Environmental Sciences Editor-in-Chief
